# First study of pathogen load and localisation of ovine footrot using fluorescence *in situ* hybridisation (FISH)

**DOI:** 10.1016/j.vetmic.2015.01.022

**Published:** 2015-04-17

**Authors:** Luci A. Witcomb, Laura E. Green, Leo A. Calvo-Bado, Claire L. Russell, Edward M. Smith, Rose Grogono-Thomas, Elizabeth M.H. Wellington

**Affiliations:** a School of Life Sciences, University of Warwick, Gibbet Hill Campus, Coventry, UK; b School of Veterinary Sciences, University of Bristol, Langford House, Langford, UK; c UCL School of Pharmacy, University College London, 29-39 Brunswick Square, London, UK; d Imerys Ltd., Par Moor Centre, Par Moor Rd, Par, Cornwall PL24, UK

**Keywords:** *Dichelobacter nodosus*, *Fusobacterium necrophorum*, Footrot, Fluorescence *in situ* hybridisation, Confocal microscopy

## Abstract

Analysis of bacterial populations *in situ* provides insights into pathogen population dynamics and potential reservoirs for disease. Here we report a culture-independent study of ovine footrot (FR); a debilitating bacterial disease that has significant economic impact on sheep farming worldwide. Disease begins as an interdigital dermatitis (ID), which may then progress to separation of the hoof horn from the underlying epidermis causing severe footrot (SFR). *Dichelobacter nodosus* is the causative agent of ovine FR, however, the role of *Fusobacterium necrophorum* and other bacteria present in the environment and on the feet of sheep is less clear. The objective of this study was to use fluorescence *in situ* hybridisation (FISH) to detect, localise and quantify *D. nodosus*, *F. necrophorum* and the domain Bacteria from interdigital skin biopsies of healthy, ID- and SFR-affected feet. *D. nodosus* and *F. necrophorum* populations were restricted primarily to the epidermis, but both were detected more frequently in feet with ID or SFR than in healthy feet. *D. nodosus* cell counts were significantly higher in feet with ID and SFR (*p* < 0.05) than healthy feet, whereas *F. necrophorum* cell counts were significantly higher only in feet with SFR (*p* < 0.05) than healthy feet. These results, together with other published data, indicate that *D. nodosus* likely drives pathogenesis of footrot from initiation of ID to SFR; with *D. nodosus* cell counts increasing prior to onset of ID and SFR. In contrast, *F. necrophorum* cell counts increase after SFR onset, which may suggest an accessory role in disease pathogenesis, possibly contributing to the severity and duration of SFR.

## Highlights

• First FISH study of ovine FR to examine pathogen spatial distribution and load.
• *D. nodosus* cell counts are significantly associated with both ID and SFR.
• *F. necrophorum* cell counts are significantly associated with SFR.
• Highlights FISH as a useful tool for studying microbial populations *in situ*.

## 1 Introduction

Ovine footrot (FR) is an infectious bacterial disease that causes lameness and affects sheep flocks worldwide (Kaler and Green, 2008; Hussain et al., 2009; König et al., 2010). Damage to the interdigital skin is thought to be required for disease to occur (Beveridge, 1941). Early stages of FR present as an inflammation of the interdigital skin (interdigital dermatitis (ID)) and later stages present with sloughing of necrotic epithelium (severe footrot (SFR)) (Beveridge, 1941; Egerton et al., 1969). In some sheep this is followed by inflammation of the epidermis below the horn that causes the horn capsule to separate from the epithelium leading to severe footrot (SFR) (Beveridge, 1941). The primary causative agent of ovine FR is *Dichelobacter nodosus* (Beveridge, 1941; Kennan et al., 2001, 2010). *Fusobacterium necrophorum* is commonly detected in FR lesions and is reported to be essential for disease initiation (Egerton et al., 1969; Roberts and Egerton, 1969). However, changes in *D. nodosus* and *F. necrophorum* populations detected by qPCR indicate that *F. necrophorum* load increases after SFR has occurred suggesting that it is an opportunistic, secondary pathogen (Witcomb et al., 2014). This is supported by a plethora of studies investigating *D. nodosus*, ranging from molecular infection trials (Kennan et al., 2001, 2010) to molecular genetic epidemiological studies correlating genotype with clinical presentation (Kennan et al., 2014; Stäuble et al., 2014).

*D. nodosus* and *F. necrophorum* have been detected using a range of culture-dependent and -independent techniques from both swabs and biopsies collected from the interdigital skin of sheep with ID and SFR (Bennett et al., 2009; Calvo-Bado et al., 2011; Witcomb et al., 2014). The advantages and disadvantages of culture-dependent and -independent methods have been discussed elsewhere (Rogers et al., 2009), but due to the fastidious nature of both anaerobes, PCR is more sensitive than culturing methods (Moore et al., 2005). Additionally problematic is the marked pleomorphism of *F. necrophorum* and other bacteria and the limited morphologies presented within a genus; making reliable identification of *F. necrophorum* and *D. nodosus* using morphology alone prone to error (Hofstad, 2006; Young, 2007). In contrast, fluorescence *in situ* hybridisation (FISH) can be used to detect bacteria in their natural environment, providing information regarding bacterial load and localisation (Baumgart et al., 2007; Amann and Fuchs, 2008). The use of specific fluorescently tagged oligonucleotide probes provides a sensitive and specific improvement on conventional light microscopy, which relies on phenotypic recognition of bacterial species. FISH has recently been used to detect *D. nodosus* and *F. necrophorum* in cases of ovine and bovine foot disease (Rasmussen et al., 2012; Klitgaard et al., 2013; Knappe-Poindecker et al., 2014) but no study to date has used FISH to investigate bacterial load and localisation patterns for cases of ID and SFR in sheep.

The aim of this study was to use FISH to investigate the spatial distribution and load of the domain Bacteria, *D. nodosus* and *F. necrophorum* populations from all four feet of six sheep (*n* = 24 biopsies) with a range of non-experimentally induced disease states (healthy (H), ID and SFR). Results demonstrate that changes in *D. nodosus* and *F. necrophorum* cell counts correlate with changes in disease state.

## 2 Methods

### 2.1 Collection of interdigital skin biopsies and swabs

A flock of 99 ewes with 146 lambs were monitored for 10 months (Smith et al., 2014). At the end of the 10-month study, six sheep were selected based on disease history (healthy *n* = 2, ID *n* = 2, SFR *n* = 2) and for those with disease, the disease severity was recorded (Foddai et al., 2012) at the time of slaughter. Interdigital swabs (*n* = 24) were collected from feet prior to biopsy punch, and chromosomal DNA extracted as previously described (Moore et al., 2005). *F. necrophorum* (*rpoB*) and *D. nodosus* (*rpoD*) amplicons were then detected and quantified by qPCR as done elsewhere (Witcomb et al., 2014).

Interdigital skin punch biopsies (*n* = 24) were collected from all four feet of these six sheep using disposable sterile Biopsy Punches (8 mm diameter) (Stiefel Laboratories, UK) immediately post mortem at the EU-licensed red meat abattoir at Bristol Veterinary School. There were *n* = 12 biopsies from healthy feet, *n* = 6 biopsies from feet with ID and *n* = 6 biopsies from feet with SFR. A total of 4/12 healthy foot biopsies came from sheep with other feet affected by ID and/or SFR, the remaining samples belonged to sheep with all feet being classified as healthy. Biopsies were fixed immediately in 3.8–4.0% (w/v) neutral buffered formalin (NBF) overnight and embedded and sectioned at Bristol Pathology Laboratory (Bristol Veterinary School, University of Bristol, Langford, UK). Collection of ovine clinical material was approved by the University of Bristol local ethical committee.

### 2.2 Fluorescence *in situ* hybridisation (FISH) probes and protocol

**Supplementary Fig. 1:**
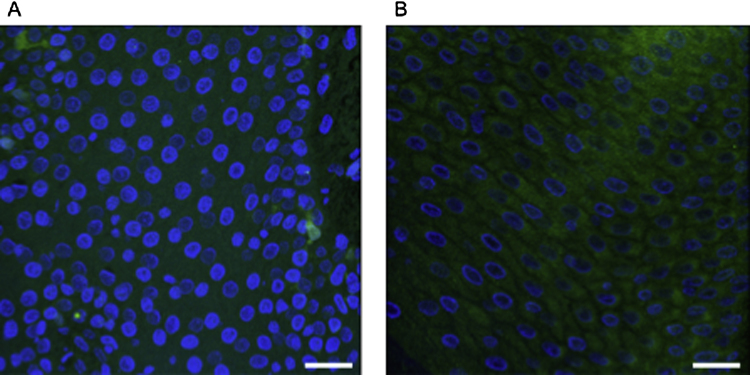
DAPI stained ovine interdigital biopsy section (A) with fluorescence *in situ* hybridisation reaction using EUK1195 probe (B). Cell junctions can be seen with EUK1195 staining. Scale bars: 5 μm.

The *D. nodosus* probe (5′-TCGGTACCGAGTATTTCTAC-3′) was modified from the Cc primer sequence (La Fontaine et al., 1993) targeting the 16S rRNA gene sequence positions 821–840 (Dewhirst et al., 1990). The *F. necrophorum*_183 probe (Boye et al., 2006), the EUB338 probe set (-I, -II, -III) consisting of three probes (Alm et al., 1996; Daims et al., 1999) and the EUK1195 probe (Giovannoni et al., 1988) were also used for this study. The EUB338-I probe covers 90% of the domain Bacteria, and EUB338-II and -III were included to extend coverage (Alm et al., 1996; Daims et al., 1999). The EUK1195 probe was used to provide definition to eukaryotic cell junctions (Supplementary Fig. 1). Bacterial probes and the EUK1195 probe were labelled at the 5′-end with Cy3 and FITC, respectively.

Supplementary Fig. 1 related to this article can be found, in the online version, at http://dx.doi.org/10.1016/j.vetmic.2015.01.022.

The FISH procedure was performed as described previously (Amann et al., 1996; Peters et al., 2011). Briefly, after dehydration steps in an ethanol series, slides were incubated with hybridisation buffers for 4 h at 46 °C. Hybridisation reactions contained 50–60 ng μl^−1^ FISH probe (*D. nodosus*, *F. necrophorum* or EUB338 probe set in conjunction with EUK1195); an equimolar mixture of the EUB338-I, -II, -III probes was used. After washing, the slides were dried using pressurised air canisters and mounted in VectaShield Mounting Medium containing 4′,6′-diamidino-2-phenylindole (DAPI) (H-1200). All tissue sections were pre-treated with proteinase K (5 μg ml^−1^) for 10 min at room temperature (20–22 °C) (Peters et al., 2011). Tissue sections to be screened with the EUB338 probe set were pre-treated with lysozyme (10 mg ml^−1^). Lysozyme pre-treatment was not required for the *D. nodosus* or *F. necrophorum* screens.

### 2.3 FISH optimisation

The *in silico* specificity of the *D. nodosus* probe was determined using probeCheck (http://www.microbial-ecology.net/probecheck) (Loy et al., 2008). A series of positive and negative control microorganisms (*n* = 14) were then screened using the *D. nodosus* oligonucleotide to assess the specificity of the probe. The microorganisms screened were selected because they had previously been detected on the ovine foot by pyro-sequencing (Calvo-Bado et al., 2011) or isolation (Nicky Buller, personal communication) or they represented environmentally relevant non-target microorganisms (Table 1). Empirical optimisations were carried out for each probe/probe set to determine specificity and optimal formamide concentrations (0–35% [v/v]) to adjust the stringency of the hybridisation. Biopsies from healthy feet were subcutaneously inoculated using sterile 25 gauge (G) needles (BD Microlance™ 3, BD, Drogheda, Ireland) with 100 μl of either *F. necrophorum* (BS-1) or *D. nodosus* (VCS1703A) at several sites to distribute the inocula evenly and incubated at 37 °C under anaerobic conditions for 1 h and 24 h to act as spiked positive controls (data not shown). Tissue controls were then screened using the relevant bacterial probe and the EUB338 probe set.

### 2.4 Confocal microscopy and image analysis

Images were obtained using a scanning confocal Leica TCS SP5 microscope (Leica Microsystems Ltd., Milton Keynes, UK) equipped with Blue Diode (405 nm), Argon gas (488 nm), Ti sapphire (561 nm) and Orange HeNe (596 nm) lasers. Pre-set narrow bandwidth settings were used to analyse the DAPI, FITC and Cy3 signals. Image processing was performed using the Leica LAS image analysis software and open source ImageJ software (http://rsb.info.nih.gov.ij) (Abramoff et al., 2004). Multiple images/fields of view (FOV) (*n* = 9–12) were taken from each foot biopsy for localisation purposes. Bacterial counts and localisation patterns were recorded per image and each image standardised to 156 μm^2^ (Davenport and Curtis, 2004; Baumgart et al., 2007); this FOV size was used to accurately identify individual bacterial cells for quantification. Bacterial counts were then +1 log_10_ transformed for downstream analysis and data expressed as log_10_ bacterial count/FOV.

### 2.5 Statistical analysis and multinomial modelling

Bacterial counts were +1 log_10_ transformed (to reduce skew) and then averaged for each biopsy. A mixed effect continuous outcome model was used to estimate log_10_ mean load of the bacteria by disease state adjusted for clustering of feet within sheep. An unordered multinomial mixed effects model accounting for clustered feet within sheep in MLwiN 2.21 software, Bristol, UK (Rasbash et al., 2005) was used to examine the associations between *D. nodosus* and *F. necrophorum* log_10_ load by disease status. The outcome variable had three categories; healthy, ID- and SFR-affected feet. The explanatory variables were *D. nodosus* and *F. necrophorum* log_10_ load. The model was built using a forward stepwise approach. The equation took the form:

$$\text{Log}(\pi_{1\text{jk}/\text{pi}0\text{jk}})=\beta_{0\text{k}}+\sum \beta_{0}x_{\text{jk}}+\sum \beta_{0}x_{\text{j}}+v_{0\text{k}}$$

$$\text{Log}(\pi_{2\text{jk}/\text{pi}0\text{jk}})=\beta_{1\text{k}}+\sum \beta_{1}x_{\text{jk}}+\sum \beta_{1}x_{\text{j}}+v_{1\text{k}}$$

where log(π_1jk/pi0jk_) = the probability of ID *versus* healthy and log(π_2jk/pi0jk_) = the probability of SFR *versus* healthy, *β*_0k_ and *β*_1k_ are constants for ID and SFR, *β*_0_*x* and *β*_1_*x* are vectors of fixed effects for ID and SFR varying at level 1 and 2, where level 1(j) = feet and level 2(k) = sheep, where *v*_0k_ and *v*_1k_ are level 2 residual variances and level 1 is assumed to take a binomial error distribution. The model was developed using RIGLS (Restricted Iterative Generalised Least Squares) and then MCMC (Markov Chain Monte Carlo) was used to adjust for the possibility of overinflated standard errors. A burn in of 5,000 followed by 50,000 iterations was done. Significance was determined using the Wald's statistic, where 95% confidence intervals (CI) did not include unity. The model fit was tested by outputting the predictions from the model and comparing sum ranked fitted quintile estimates against the summed observations for the number of cases of ID and SFR combined each week using the Hosmer Lemeshow test (Doohoo et al., 2003).

## 3 Results

### 3.1 *In vitro* and *ex vivo* optimisation of FISH protocol

The *D. nodosus* oligonucleotide probe was determined to be specific and produce the highest signal-to-noise ratio with 25% formamide in the hybridisation buffer (data not shown), with no binding to non-specific microorganisms observed (Table 1). In addition, the FISH protocols were tested empirically both *in vitro* and on tissue biopsies spiked with *D. nodosus* or *F. necrophorum* cells (data not shown).

### 3.2 Tissue observations and detection of bacterial populations from interdigital swabs and biopsies

**Fig. 1:**
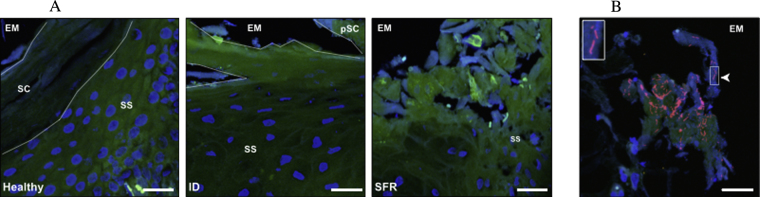
Tissue morphology of biopsy sections by disease state (healthy, ID and SFR) (A), and evidence of sloughing of necrotic tissue carrying bacterial cells (B). Bacterial cells (red), epithelial cells (green), epithelial cell nuclei (blue) and erythrocytes (white – autofluorescence). *Stratum corneum* (SC), partial *stratum corneum* (pSC), *stratum spinosum* (SS) and extracellular milieu (EM) are shown (red channel images removed from (A) for tissue morphology to be observed). Scale bars: 25 μm. (For interpretation of the references to colour in this figure legend, the reader is referred to the web version of this article.)

**Fig. 2:**
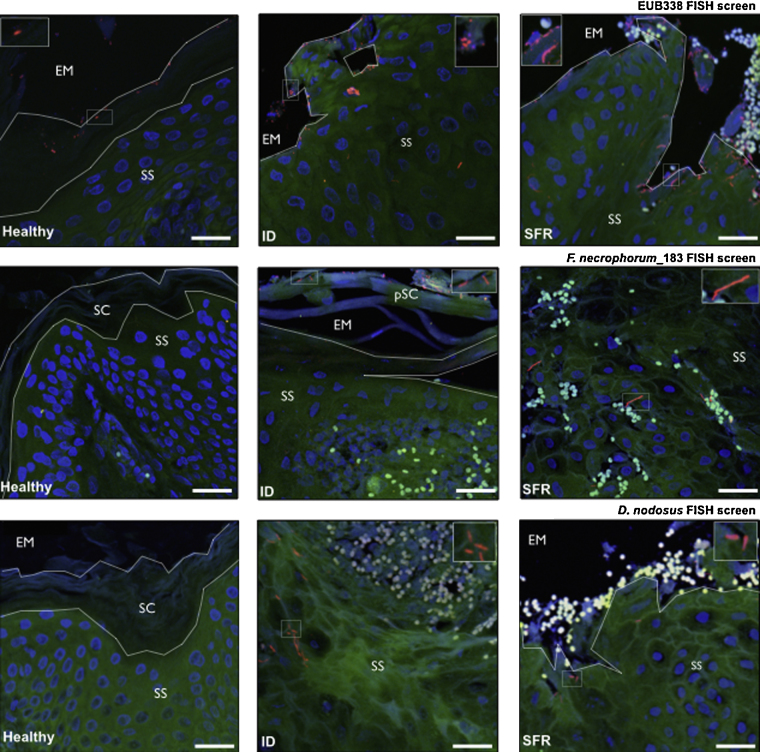
Representative FISH images from biopsy sections by disease state (healthy, ID and SFR). Bacterial cells (red), epithelial cells (green), epithelial cell nuclei (blue) and erythrocytes (white – autofluorescence). *Stratum corneum* (SC), partial *stratum corneum* (pSC), *stratum spinosum* (SS) and extracellular milieu (EM) are shown. Scale bars: 25 μm. (For interpretation of the references to colour in this figure legend, the reader is referred to the web version of this article.)

For healthy feet, 1/12 and 6/12 swabs were positive for *F. necrophorum* (*rpoB*) and *D. nodosus* (*rpoD*), respectively. Samples that were positive were on the limit of detection (∼10^3^ copies swab^−1^), which is consistent with earlier findings (Calvo-Bado et al., 2011; Witcomb et al., 2014). A total of 5/6 and 6/6 swabs from ID feet were positive for *F. necrophorum* (*rpoB*) and *D. nodosus* (*rpoD*), respectively. Similarly, 100% of swabs were positive for both bacterial species from feet with SFR. Significant necrosis of the *stratum corneum* was present in tissue from feet with ID and SFR and bacterial cells were observed in the sloughed necrotic tissue, sometimes in large numbers (Fig. 1). In addition, the infiltration of erythrocytes was associated with both stages of disease and absent in healthy feet (Fig. 2). Erythrocytes appeared as auto-fluorescent cells under all three channels.

**Supplementary Fig. 2:**
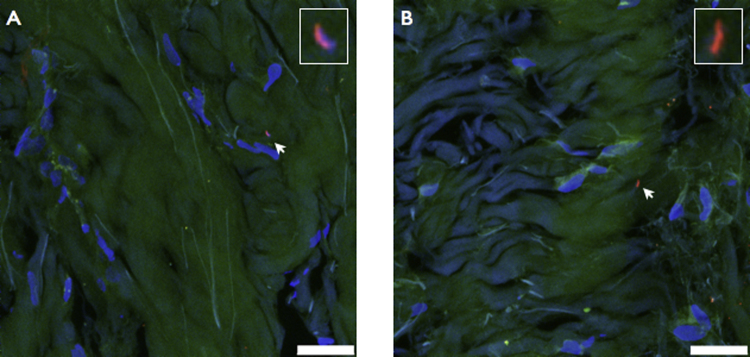
Detection of *D. nodosus* (A) and *F. necrophorum* (B) within dermal layers of interdigital skin biopsies. Scale bars: 5 μm.

EUB338-I, -II, -III labelled cells were detected on the surface of or within the epidermis of 100% of biopsies (24/24), acting as a positive FISH control (Moter and Göbel, 2000). *F. necrophorum* cells were detected in 1/12, 4/6 and 5/6 of H, ID and SFR interdigital skin biopsies, respectively. In contrast, *D. nodosus* cells were not detected in biopsies from healthy feet, whether other feet from the sheep were diseased or not (*n* = 0/12), but were detected in 50% of biopsies from feet with ID (*n* = 3/6) and SFR (*n* = 3/6). The vast majority of microorganisms detected by FISH were located in the epidermis, which is consistent with other work (Egerton et al., 1969; Rasmussen et al., 2012). However, one *F. necrophorum* cell and one *D. nodosus* cell were detected in the dermis of two different biopsies (Supplementary Fig. 2).

Supplementary Fig. 2 related to this article can be found, in the online version, at http://dx.doi.org/10.1016/j.vetmic.2015.01.022.

Fluorescently tagged bacterial cells were counted in individual FOVs for the epidermis (*n* = 3–4) from each biopsy per oligonucleotide probe. Quantification was limited to images of the epidermis, as *D. nodosus* and *F. necrophorum* populations were primarily restricted to this region. Bacterial cell counts from images were +1 log_10_ transformed and compared by disease state (H, ID and SFR) using a mixed effect model accounting for clustering of feet by ewe (Table 2). Representative images are shown in Fig. 2. Mean cell counts for the domain Bacteria were not significantly different (*p* > 0.05) between disease states. In contrast, *D. nodosus* cell counts were significantly higher in both feet with ID and SFR than healthy feet (*p* < 0.05) and *F. necrophorum* cell counts were only significantly higher in feet with SFR than healthy feet (*p* < 0.05).

## 4 Discussion

This study used FISH to detect, quantify and localise *D. nodosus* and *F. necrophorum* populations from biopsies collected from healthy, ID- and SFR-affected ovine interdigital skin.

The key novel result was that whilst the general bacterial population remained relatively stable by disease state, *D. nodosus* and *F. necrophorum* populations changed with clinical presentation. *F. necrophorum* cell counts were significantly higher in cases of SFR (*p* < 0.05), whilst *D. nodosus* cell counts were significantly higher in cases of ID and SFR than in healthy feet (*p* < 0.05); the model accounted for dependencies between feet within sheep (Table 2). These findings suggest a shift in the *D. nodosus* and *F. necrophorum* populations within the interdigital skin before ID and between ID and SFR and these data are consistent with Calvo-Bado et al. (2011) and Witcomb et al. (2014) who both reported that *D. nodosus* (*rpoD*) load was highest before and during an episode of ID and before SFR by qPCR and that *F. necrophorum* (*rpoB*) load instead increased after SFR (Witcomb et al., 2014). The findings from the current study therefore provide further evidence supporting Witcomb et al. (2014) who proposed that an increase in *D. nodosus* population numbers drives pathogenesis of footrot from healthy to ID to SFR whilst *F. necrophorum* population increased only after SFR developed. The causal role of *D. nodosus* is further supported by a number of genomic and epidemiological studies, which correlate *D. nodosus* genotype with clinical presentation (Kennan et al., 2001, 2010, 2014; Stäuble et al., 2014).

The bacterial populations in the ovine interdigital biopsies were primarily restricted to the superficial epidermal layers, consistent with previous reports (Egerton et al., 1969; Rasmussen et al., 2012). The presence of *D. nodosus* by FISH (50% ID and SFR biopsies) was lower than that detected by qPCR studies, where *D. nodosus* DNA (*rpoD*) was detected in 86% and 71% of swabs from feet with ID and SFR, respectively (Witcomb et al., 2014). The FISH data are more in-line with isolation results; with *D. nodosus* detected in 67.9% and 55.8% swabs from feet with ID and SFR, respectively, and not detectable in samples from healthy feet (Moore et al., 2005). We postulate that this is due to the higher sensitivity of the qPCR assays compared with FISH, which is supported by the increased detection frequency of *rpoB*/*rpoD* amplicons from the interdigital skin. The swab samples gather superficial bacteria from a wide area of the interdigital skin, whilst the biopsy only samples an 8 mm region of the epidermis. *D. nodosus* may not have been present in the samples collected, however, we feel this is unlikely considering the qPCR data and the strong association with disease. Whilst *F. necrophorum* was detected in most biopsies of ID and SFR, load was not significantly associated with ID, consistent with findings elsewhere (Witcomb et al., 2014). In addition, a larger bacterial population (as represented by load or cell count) may indicate a longer established community, suggesting *D. nodosus* preceded *F. necrophorum* in cases of ID. Other studies that have reported determining the presence/absence of *F. necrophorum* have also reported an increased detection frequency of *F. necrophorum* in FR (Bennett et al., 2009), and concluded that this organism therefore likely contributes to the pathogenesis of FR. In contrast, Witcomb et al. (2014) found that both *F. necrophorum* and *D. nodosus* are frequently present, even in healthy feet, and therefore examining bacterial load is more informative than the presence/absence in studies of causality.

Erythrocytes were present in diseased tissue sections, indicative of damage to local capillaries and seepage of blood out from leaky capillary walls. There was also evidence of sloughing of epidermal tissue in sections from diseased biopsies and the tissue included bacterial cells; which might indicate a route of transfer of infection between diseased and susceptible feet and sheep (Beveridge, 1941). *D. nodosus* and *F. necrophorum* cells were detected within the dermal layers in two separate biopsies. It is possible that these were artefacts produced during processing of tissue samples, however, once the tissues are fixed, their architecture is maintained (Hopwood, 1991) and so the formation of artefacts affecting bacterial localisation seems unlikely. If this observation is a true finding, it would suggest that these anaerobes are able to penetrate the deeper dermal layers on occasion, which may act as a potential reservoir for chronic infection. Egerton et al. (1969) also reported that *F. necrophorum* was present in the dermis of ovine feet in cases of FR.

The small sample size is a limitation of this study, which may have implications for interpretation of results, however, the data are consistent with a number of other studies using a variety of culture-dependent and -independent methodologies (Moore et al., 2005; Calvo-Bado et al., 2011; Witcomb et al., 2014). Ideally longitudinal sampling would also have been performed to follow disease progression over time in the same individual, however punch biopsies are invasive and taken over time would likely predispose sheep feet to infection and change the natural progression of disease.

In conclusion, this study describes in detail the detection, spatial distribution and quantification of *D. nodosus*, *F. necrophorum* and the domain Bacteria within ovine interdigital skin biopsies and their association with healthy, ID and SFR using FISH. We also present evidence that bacterial cell counts change with clinical presentation, with *D. nodosus* counts significantly higher in ID and SFR and *F. necrophorum* cell counts increasing only after progression to SFR. This is consistent with previous work (Calvo-Bado et al., 2011; Witcomb et al., 2014) indicating that *D. nodosus* initiates ID and is present before SFR develops, whilst *F. necrophorum* cell counts only increase when SFR is present. Finally, this study supports FISH as an invaluable tool that can be used to examine the microbial community associated with ovine FR.

## Funding

L.A. Witcomb was a NERC CASE studentship with Pfizer Animal Health as the industrial partner. All other authors were supported by Combating Endemic Diseases of Farmed Animals for Sustainability (CEDFAS) initiative, Grant No. BBE01870X1 from the Biotechnology and Biological Sciences Research Council (BBSRC).

## Figures and Tables

**Table 1:** Specificity of *D. nodosus* oligonucleotide probe (modified from the Cc forward primer) (La Fontaine et al., 1993). Binding conditions altered by increasing formamide within the hybridisation buffer.

*D. nodosus* strain (positive controls)	Result	Negative controls	Result
VCS1703A	+	*Aeromonas hydrophila*^a^	−
BS-1	+	*Aeromonas media*^a^	−
BS-6	+	*Arcanobacterium pyogenes* (DS7M 20–630)	−
A198	+	*Bacillus circulans* (WL-12)^b^	−
Serogroup A	+	*Citrobacter freundii*^a^	−
		*Escherichia coli* (K12)	−
		*Fusobacterium necrophorum* (BS-1)	−
		*Klebsiella pneumoniae*^a^	−
		*Macrococcus caseolyticus*^c^	−

a Environmental isolate (river water), United Kingdom.

b Environmental isolate (soil), United Kingdom.

c Bovine isolate (milk), United Kingdom.

**Table 2:** Three mixed effects regression models providing log_10_ mean cell counts/FOV for the domain Bacteria (EUB338), *D. nodosus* (Dn) and *F. necrophorum* (Fn) in feet with ID (*n* = 6) and SFR (*n* = 6) compared with a baseline of healthy feet (*n* = 12).^a^

		Log_10_ mean	s.e.	Lower 95% CI	Upper 95% CI
EUB338	Healthy/baseline	1.074	0.144	0.792	1.356
	ID	0.305	0.250	−0.185	0.795
	SFR	0.283	0.251	−0.209	0.775

Dn	Healthy/baseline	−0.023	0.142	−0.301	0.255
	**ID**	**0.412**	**0.187**	**0.045**	**0.779**
	**SFR**	**0.469**	**0.215**	**0.0476**	**0.8904**

Fn	Healthy/baseline	0.006	0.113	−0.215	0.227
	ID	0.321	0.195	−0.061	0.703
	**SFR**	**0.714**	**0.195**	**0.332**	**1.096**

ID: interdigital dermatitis, SFR: severe footrot, s.e.: standard error, CI: confidence interval, bold: *p* < 0.05.

a Adjusted for repeated measures within feet and sheep.
